# RNA sequencing and *de novo* assembly of the digestive gland transcriptome in *Mytilus galloprovincialis* fed with toxinogenic and non-toxic strains of *Alexandrium minutum*

**DOI:** 10.1186/1756-0500-7-722

**Published:** 2014-10-14

**Authors:** Marco Gerdol, Gianluca De Moro, Chiara Manfrin, Anna Milandri, Elena Riccardi, Alfred Beran, Paola Venier, Alberto Pallavicini

**Affiliations:** Laboratory of Genetics, Department of Life Sciences, University of Trieste, Via Licio Giorgeri 5, Trieste, 34126 Italy; Fondazione Centro Ricerche Marine, viale Amerigo Vespucci 2, Cesenatico, Forlì-Cesena 47042 Italy; Dipartimento di Oceanografia Biologica, Istituto nazionale di Oceanografia e di Geofisica sperimentale, via Auguste Piccard 54, Santa Croce, Trieste 34151 Italy; Department of Biology, University of Padua, Via Bassi 58 / B 35121, Padova (PD) Padua, Italy

**Keywords:** Mytilus galloprovincialis, Paralytic shellfish poisoning, De novo assembly, Alexandrium minutum, RNA-seq, transcriptome

## Abstract

**Background:**

The Mediterranean mussel *Mytilus galloprovincialis* is marine bivalve with a relevant commercial importance as well as a key sentinel organism for the biomonitoring of environmental pollution. Here we report the RNA sequencing of the mussel digestive gland, performed with the aim: a) to produce a high quality *de novo* transcriptome assembly, thus improving the genetic and molecular knowledge of this organism b) to provide an initial assessment of the response to paralytic shellfish poisoning (PSP) on a molecular level, in order to identify possible molecular markers of toxin accumulation.

**Results:**

The comprehensive *de novo* assembly and annotation of the transcriptome yielded a collection of 12,079 non-redundant consensus sequences with an average length of 958 bp, with a high percentage of full-length transcripts. The whole-transcriptome gene expression study indicated that the accumulation of paralytic toxins produced by the dinoflagellate *Alexandrium minutum* over a time span of 5 days scarcely affected gene expression, but the results need further validation with a greater number of biological samples and naturally contaminated specimens.

**Conclusion:**

The digestive gland reference transcriptome we produced significantly improves the data collected from previous sequencing efforts and provides a basic resource for expanding functional genomics investigations in *M. galloprovincialis*. Although not conclusive, the results of the RNA-seq gene expression analysis support the classification of mussels as bivalves refractory to paralytic shellfish poisoning and point out that the identification molecular biomarkers of PSP in the digestive gland of this organism is problematic.

**Electronic supplementary material:**

The online version of this article (doi:10.1186/1756-0500-7-722) contains supplementary material, which is available to authorized users.

## Background

The advent of next generation sequencing has definitely expanded large-scale molecular studies to non-model organisms, including marine invertebrates [1]. Based on Next Generation Sequencing (NGS) technologies, the massive analysis of bivalve transcriptomes by RNA-sequencing (RNA-seq) is progressively revealing the molecular basis of the functional responses to environmental changes [2], and paving the way to an improved view of the evolutionary relationships among mollusks [3, 4]. Furthermore, the recent release of the first fully sequenced bivalve genomes represented a real milestone in mollusk genomics, offering new resources for large-scale comparative studies [5, 6].

Although the knowledge of genes expressed in mussels (*Mytilus* spp.) substantially improved in recent years, starting with massive ESTs sequencing efforts [7] and moving towards modern pyrosequencing approaches [8–12], the current view of mussel transcriptome is still fragmentary, due to the limited sequencing depth applied so far and to the error rate of 454 pyrosequencing, factors which both prevent a good reconstruction of poorly expressed full length transcripts.

Besides the undeniable usefulness of RNA-seq as a tool for *de novo* transcriptome assembly and analysis, such approach also provides a far more precise gene expression measurement than other methods [13], overcoming most of the technical limitations of cDNA microarrays which have been used for quite a long time as the gold standard for large scale gene expression studies in most organisms, including mussels [14]. RNA-seq has already been successfully applied to the study of important processes in bivalves, including immunity [9, 15, 16], sex-specific regulation [17], gametogenesis [18], larval development [19] and shell mineralization [20] and it has the potential to disentangle also complex responses to stress factors, such as those caused by global climate changes, pollutants and pathogens affecting farmed bivalves.

Based on an Illumina paired-end sequencing strategy, we report the sequencing and *de novo* assembly of the digestive gland (DG) transcriptome of the Mediterranean mussel *Mytilus galloprovincialis*. The new data significantly enrich the overall understanding of the mussel transcriptome, with a focus on a tissue known to accumulate and transform phycotoxins and pollutants, hence relevant for toxicogenomic studies. We then highlight the potential usefulness of the resulting reference transcriptome by exploring the case study of the mussel response to Paralytic Shellfish Poisoning (PSP) through the comparison of the transcription profiles of DG samples from animals fed with toxigenic or non-toxigenic strains of the dinoflagellate *Alexandrium minutum* over 5 days.

PSP is a syndrome associated with the consumption of filter-feeding mollusks contaminated with toxins usually produced by various unicellular algae, including *A. minutum* [21]. Filter-feeding organisms such as bivalve mollusks can accumulate paralytic shellfish toxins (PSTs) at very high concentrations and act as lethal vectors of toxins for organisms at higher trophic levels, including humans. The symptoms of intoxication in humans are mainly of a neurological nature [22] and are linked to the high affinity of paralytic shellfish toxins (PSTs) to the neuronal voltage-gated sodium ion channels, which results in the block of action potentials [23]. Besides representing an emerging issue for human health worldwide [24], PSP also causes severe economic damage to aquaculture because of the closure of farming areas affected by algal blooms [25, 26].

Likewise humans and other vertebrates [27–30], certain bivalve species suffer the paralytic effect of PSP, whereas others seem to be completely unaffected [31]. Although different species display different behavioral responses to PSP blooms, there is a negative relationship between the susceptibility to PSTs and the ability to feed on toxigenic algae and, consequently, to bioaccumulate toxins [31, 32]. Mussel nerves are insensible to the paralytic effects of saxitoxin (STX) [33, 34] and due to the lack of physiological and behavioral changes in response to the feeding with PSP-producing dinoflagellates, *Mytilus* spp. are generally considered refractory to PSP and can therefore accumulate toxins in their tissues (mainly in the digestive gland) at high concentrations [35]. On the other hand, other reports seem to indicate that mussels can be occasionally negatively influenced by PSTs, in terms of increased mortality, histopathological modifications and decreased filtration rate [36–39]. A few recent works have tried to elucidate the molecular response of bivalves to Harmful Algal Blooms (HABs) based on gene-focused or proteomic analyses [24, 40]. Nevertheless, the only two whole-transcriptome studies on the effects of phycotoxins performed to date in mussels concerned okadaic acid, and provided a significant improvement upon the understanding of the molecular effects of this compound in *M. galloprovincialis* [10, 41]. Using similar methodologies, the detection of molecular changes occurring in response to PSTs accumulation could reveal which strategy, if any, mussels adopt to cope with significant amounts of bioaccumulated toxins.

Overall, the analysis we performed did not reveal a specific transcriptional pattern of response to PSP in the mussel DG, in agreement with most of the physiological data collected so far and with the classification of mussels as organisms refractory to PSP. On the other hand, due to the low number of samples analyzed these data have to be considered as preliminary, and certainly need to be confirmed by taking into account a greater number of biological samples. Nevertheless, we identified a limited number of potential biomarkers of PSP contamination, which will require further experimental validation on naturally contaminated samples.

## Results

### High throughput sequencing of the digestive gland transcriptome

The Illumina sequencing of the DG samples, generated 57,059,700 paired-end reads. The average read length was 94.5 bp, overall equivalent to ~5.4 Gbp of sequence. Table 1 summarizes the trimming statistics and the number of sequenced reads per sample. Overall, approximately 7.5% of the sequenced reads did not pass the quality control check and were discarded prior to the assembly. The raw Illumina reads generated for this experiment were deposited at the NCBI Sequence Read Archive (SRA: SRP011280.2).

**Table 1:** **RNA-sequencing and** ***de novo*** **assembly statistics**

Trimming statistics		
Number of reads before trimming		57,059,700
Number of reads after trimming		52,770,704
Sequences discarded during trimming		7.52%
Average length before trimming		94.5 bp
Average lenth after trimming		97.3 bp
**Number of reads per sample**		
T1 non-toxic strain-fed (AL1T)		6,102,912
T1 toxic strain-fed (AL9T)		14,571,224
T2 non-toxic strain-fed (AL1T)		16,419,080
T2 toxic strain-fed (AL9T)		15,678,278
**Additional sequences used for the assembly**		
Illumina (digestive gland)		49,871,662
454 (various tissues)		115,557
Sanger (various tissues, Mytibase collection)		18,788
**Trinity assembly statistics**	**all contigs**	**longest transcript per gene**
Assembly size	16,350,006 bp	11,571,682 bp
Total number of contigs	21,193	12,079
Mapping rate	59.04%	52.00%
Non-specific matches	4.29%	0.00%
N50	1,010	1,216
Mean contig length	771 bp	958 bp
Longest contig	14,931 bp	14,931 bp
**Annotation statistics (longest transcript per gene model)**		
Contigs with BLAST hit vs UniProtKB/Swiss-Prot		5,818 (48.1%)
Contigs with BLAST hit vs *C. gigas* predicted proteins		7,227 (59.8%)
Contigs with BLAST hit vs *P. fucata* predicted proteins		6,943 (57.5%)
Contigs with BLAST hit vs *L. gigantea* predicted proteins		6,699 (55.5%)
Contigs with InterPro domains		5,432 (45.0%)
Contigs with PFAM domains		5,696 (47.2%)
Contigs with eggNOG terms		4,524 (37.5%)
Contigs with GO Cellular Component terms		4,920 (40,7%)
Contigs with GO Biological Process terms		4,207 (34.8%)
Contigs with GO Molecular Fuction terms		4,236 (35.1%)

### Overall *de novo*assembly of the mussel digestive gland transcriptome

The raw *de novo* Trinity assembly of all the available sequencing reads generated 21,193 contigs satisfying the minimum quality criteria specified in the materials and methods section, with an average length of 771 bp for a total of 16.35 Mbp. The non-redundant contigs set, comprising only the longest isoform per each gene, used as a high-quality reference for the downstream analyses, comprised 12,079 contigs, characterized by remarkably higher average length (958 bp) and N50 statistics (see Table 1). 52 out of these contigs were over 5 Kb in length, with the longest one reaching 14,931 nucleotides. The removal of transcript variants, possibly originated by alternative splicing and paralogous genes, did not lead to a dramatic reduction in the reads mapping rate, which only dropped by ~7%. On the other hand, this process led to the complete elimination of redundant sequences, as the rate of multiple mappings was reduced from 4.29 to 0%.

**Figure 1:**
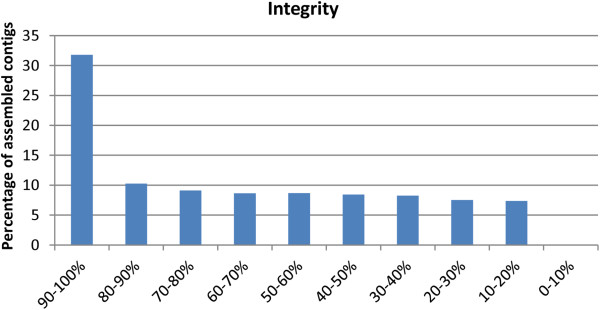
**Transcripts integrity plot.** The transcripts integrity analysis is based on BLASTx similarities of the *M. galloprovincialis* digestive gland contigs with the ortholog proteins predicted from the fully sequenced genome of *Crassostrea gigas* (with a cut-off p-value of 1x10^−5^). Ortholog coverage is displayed on the X axis, the percentage of contigs falling into each integrity category are shown on the Y axis.

Overall, we obtained a rather good reconstruction of full-length transcripts (see Figure 1): over one third of the transcripts showing a BLAST similarity to oyster proteins were assembled to their full length in respect with their orthologs, and this measure is known to be strongly under-estimated due to phylogenetic sequence divergence between species and due to the different substitution rates observed among genes [42].

### Sequence annotation

About a half (48.1%) of the contigs included in the non-redundant reference transcriptome showed similarity with protein sequences deposited in the UniProtKB/Swiss-Prot database. A slightly higher number of contigs showed similarity with proteins predicted from the fully sequenced genomes of the bivalves *C. gigas* (59.8%) [6] and *P. fucata* (57.4%) [5] and of the gastropod *L. gigantea* (55.5%). Overall, ~4,000 sequences did not find any significant match in any of the above mentioned databases (see Additional file 1 for details). Virtually no contig displayed high similarity with Prokariotes, highlighting that the transcriptome assembly was free of sequences originated from bacterial symbionts or pathogens. Similarly, the mapping of *A. minutum* NGS 454 reads from Stüken and colleagues [43] revealed only a single algal sequence present within the reference set (photosystem II protein D1), which was subsequently removed prior to the following analyses, evidencing that only a negligible portion of residual mRNA resulting from digested *A. minutum* cells was extracted from the mussel DG.

InterPro and PFAM domains could be assigned to approximately 45% of the contigs and Gene Ontology (GO) terms could be assigned to 5,508 contigs (45.6%). More in detail 4,920 were mapped to a cellular component, 4,207 to a biological process and 4,236 to a molecular function. EggNOG terms were assigned to 4,524 contigs (37.5% out of the total).

### Toxin accumulation

According to the HLPC analyses, the *A. minutum* strain AL9T produced an average concentration of 76.4 fg STXdiHCleq\cell, whereas the strain AL1T did not produce any toxins, as expected. The estimate of toxin bioaccumulation was performed on the soft mussel tissues, after the DG was taken apart for RNA extraction. PSTs, whose levels was measured in 3 individuals, were detected already at T1 and reached a concentration of about 100 μg STX eq/kg of meat at T2 (5 days from the start of the experiment). Visceral organs are largely documented as the main site of accumulation of PSTs in bivalves; however, different values have been reported in literature, depending on the species and on the time of exposure, ranging from 78 to 96% of the total toxicity [35, 44–46]. Based on these uncertainties and considering the removal of the DG, the accumulation of PSTs at T2 in the whole mussel body could be estimated to be comprised between 1,600 and 11,000 μg STX eq kg^−1^ of meat, well above the EU and US limits (set at 800 μg STX eq kg^−1^).

### RNA-seq expression analysis

**Figure 2:**
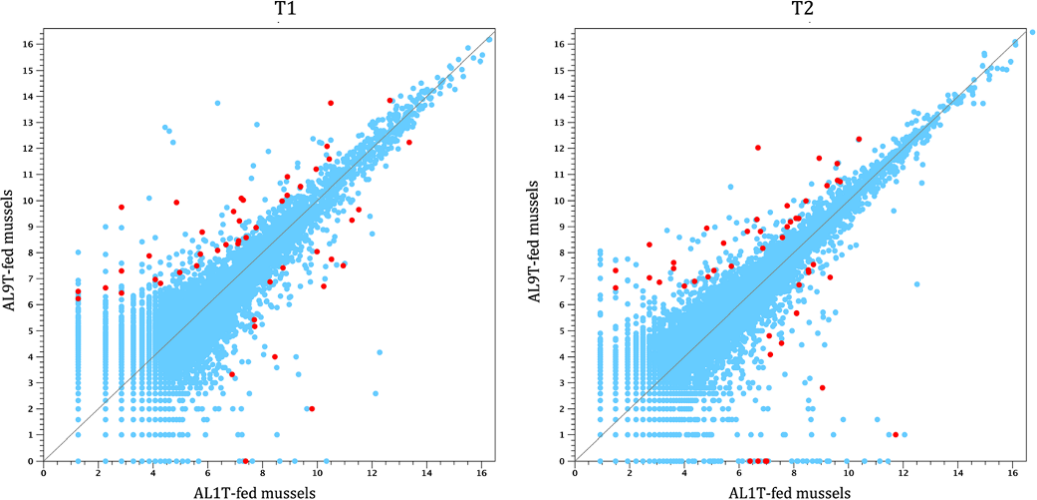
**Gene expression profiles at the early (T1) and late (T2) phase of PSP contamination.** Scatter plots displaying gene expression profiles at T1 and T2; gene expression values (displayed as log_2_ normalized read counts) are plotted for the AL1T-fed (X-axis) and AL9T-fed (Y-axis) samples. Genes identified as putative PSP biomarkers by the Kal’s Z-test on proportions at both time points are highlighted.

The differential expression analysis identified a total of 39 transcripts differentially expressed and with the same trend (up- or down-regulation) common to the two comparisons (mussels fed with the toxigenic strain AL9T vs mussels fed with the non-toxigenic strain AL1T at T1 and T2). More in detail, 28 transcripts were down-regulated and 11 were up-regulated in response to toxin accumulation. These sequences were considered as putatively responsive to PSP accumulation in the DG both at an early (24 hours) and at a late (5 days) phase of contamination and thus regarded as potential useful molecular biomarkers. The complete list of the differentially regulated transcripts is reported in Table 2. Scatter plots highlighting the differential expression of PSP-responsive transcripts in the two time points are exemplified in Figure 2.

**Table 2:** **List of putative PSP-responsive genes**

UPREGULATED		
Description	Fold change	
	T1	T2
Unknown	∞	∞
Unknown	∞	∞
BRICHOS domain-containing protein	∞	∞
Defensin-like protein	∞	∞
Peptydil-glycine alpha-amidating monooxygenase-like	∞	16.23
IMAP GTPase family member	∞	∞
Unknown	∞	∞
Zona pellucida domain containg protein	∞	∞
C-type lectin	119.40	17.62
Unknown	37.80	6.63
Zona pellucida domain containg protein	33.60	3.42
Unknown	31.15	13.79
Putative lincRNA	21.75	20.06
Hemicentin-like	20.77	57.35
Perlucin-like protein	16.16	36.09
FREP	12.05	4.65
Ganglioside GM2 activator	9.51	3.98
2'-5' oligoadenylate synthetase	7.37	48.52
Unknown	6.62	6.24
Putative lincRNA	6.21	2.50
Unknown	5.84	5.81
Unknown	4.80	13.91
Unknown	4.18	2.50
Cannabidiolic acid synthase-like	3.29	3.60
Metridin-like protein	3.12	4.15
Glycoside hydrolase	2.49	4.19
Unknown	2.41	2.39
Lysozyme	2.36	2.06
**DOWNREGULATED**		
**Description**	**Fold change**	
	T1	T2
IMAP GTPase family member	-∞	−123.60
Unknown	-∞	-∞
Ganglioside GM2 activator	-∞	-∞
Unknown	-∞	−2.68
Cadherin-like protein	-∞	-∞
Unknown	−167.11	-∞
Unknown	−21.95	−5.32
C1qDC protein	−5.85	−8.20
Laccase-like protein	−4.85	−2.27
Unknown	−2.65	−8.04
C1qDC protein	−2.53	−2.23

## Discussion

### Transcriptome richness and contigs integrity assessment

The c-value of *Mytilus galloprovincialis* genome is estimated to be comprised between 1.41 and 1.92 [47, 48], leading other authors to calculate as ~15,000 a plausible number of coding genes in this species [8]. Nevertheless, the recent sequencing of the slightly smaller genomes of *C. gigas* [6] and *P. fucata* [5] identified 28,027 and 23,257 predicted gene models respectively, thus suggesting that the mussel genome could harbor a similar, or even higher, number of genes.

Taking into consideration these estimates, the transcriptome assembly we produced, comprising ~12,000 non-redundant contigs, certainly only partially covers the genes of *M. galloprovincialis*, but likely provides a reliable snapshot of those expressed in the digestive gland. Most of the transcriptional activity in this organ is involved in the synthesis of a limited set of highly expressed messenger RNAs, since about 150 contigs of the reference assembly account for 50% of the total transcription. We applied rather stringent parameters during the assembly process, ensuring that all the contigs included in the reference transcriptome were supported by high coverage, aiming at the assembly of a high proportion of full-length transcripts. Although the integrity analysis based on the ortholog coverage revealed that about a half of the contigs were assembled to their full length or very close to it, and this can be safely considered as an under-estimation of the correct value due to inter-species divergence [42] (Figure 1), a relevant proportion of the reference transcription assembly appears to be composed by 3’ or 5’ transcript ends or by internal fragments. Since this relatively high frequency of incomplete transcripts cannot just be explained by local low coverage regions, other factors contributing to sequence fragmentation during the assembly process have to be taken into consideration, namely the occurrence of alternative splicing events, the presence of highly similar paralogous genes in the mussel genome and, most likely, the inter-individual variability [49, 50] linked to the multi-individual origin of the sequences used for the *de novo* assembly. Indeed over 9,000 contigs were assigned by the Trinity assembly algorithm to the same gene model and can therefore be counted as alternatively spliced isoforms. A more in depth analysis of the assembly output revealed that over 2,000 mussel genes produced at least 2 isoforms and almost 1,000 mussel genes produced at least five isoforms, but we could also observe cases of genes reaching as much as 50 different isoforms, revealing a remarkable transcriptomic complexity. We also briefly investigated the potential impact of inter-individual allelic variability on our assembly, identifying a total of 62,325 single nucleotide variations (SNVs), 1,028 deletions and 936 insertions over 3,539 assembled contigs. This data is not surprising, considering the extremely high level of heterozigosity observed in mussels [51]. Taken together, these data point out a significant transcriptomic complexity in mussel, likely given by high inter-individual variability, massive presence of paralogous genes and alternatively splicing isoforms, which could have negatively influenced full-length transcripts assembly and which could possibly hamper future attempts at assembling the mussel genome. Nevertheless, only its complete sequencing and annotation and the simultaneous analysis of RNA-seq data obtained from multiple geographical locations will permit a thorough investigation on these topics.

### A new important resource for mussel genomics

Although efforts have been made in the past for the generation of the transcriptomic knowledgebase of *M. galloprovincialis* Mytibase, comprising 7,112 contigs from multiple tissues, this EST database was severely affected by the technical limitations of Sanger sequencing [7]. Most recent approaches involved the RNA pyrosequencing of various tissues of the Mediterranean mussel [8, 10], and of the closely related species *Mytilus edulis* [9], with a much higher coverage. While these approaches drastically increased the sequence data available for mussel, especially for *M. edulis*, they suffered from the limitations of early 454 sequencing, characterized by rather low coverage, relatively high error rates and poor full length reconstruction compared to the potential of the Illumina paired-end sequencing.

**Figure 3:**
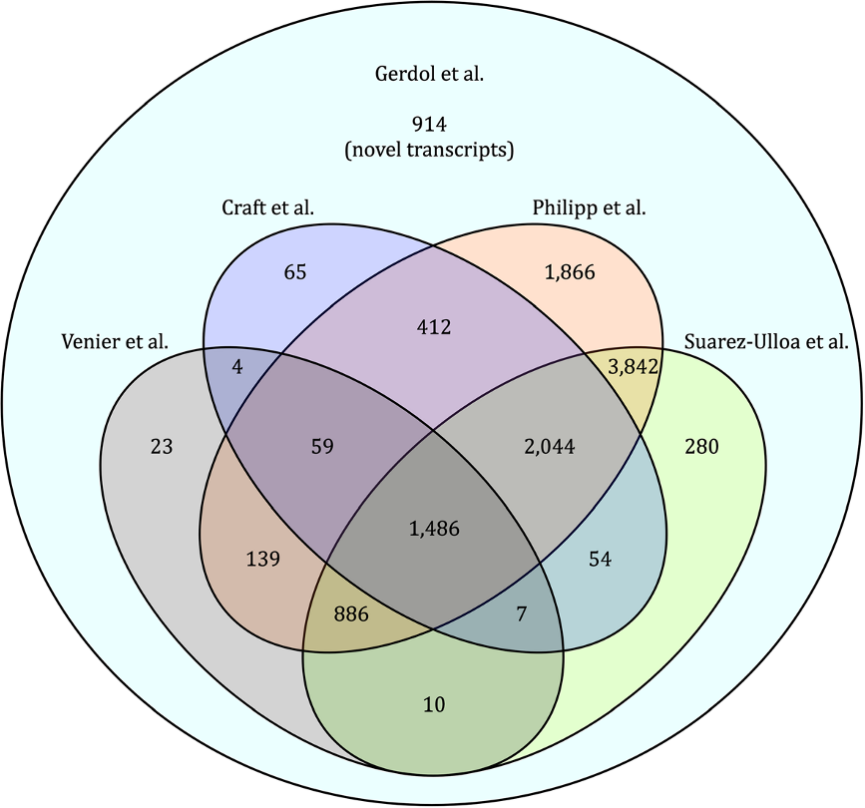
**Comparison between the recent sequencing approaches in** ***M. galloprovincialis.*** Venn diagram summarizing the overlap between the *M. galloprovincialis* reference transcriptome generated in the present work, Mytibase (Venier et al., 2009) and the pyrosequencing datasets produced by Craft et al. (2010), Philipp et al. (2012) and Suarez-Ulloa et al. (2013). Numbers shown in the graph correspond to the number of contigs showing a positive mapping hit in each sequencing set.

Although the digestive gland-focused sequence data generated by RNA-seq in the present study provide a limited view of the entire complement of transcripts expressed in different tissues, different life stages and in response to many biotic and abiotic stimuli in *M. galloprovincialis*, we expected to obtain a good coverage for a broad range of transcripts due to the high sequencing depth applied. We assessed to what extent the RNA-seq of the DG extended the available mussel sequence datasets by analyzing the mapping of Sanger and 454 sequencing reads on our non-redundant transcriptome assembly. An overview of the overlap between the different approaches provided by Venier et al. [7], Craft et al. [8], Philipp et al. [9] and Suarez-Ulloa et al. [10] is provided in Figure 3. A total of 1,486 contigs, which likely comprise housekeeping and other broadly expressed genes, are common to the five sequence datasets, but most of the digestive gland contigs were only sequenced, as expected, in the most recent 454 approaches. In particular the *M. edulis* RNA-sequencing, using rather high depth and investigating multiple tissues, appears to be the most complete. Globally, 914 contigs included in our novel digestive gland assembly did not find any match in any of the previous sequencing efforts, thus representing novel transcripts. Furthermore, our transcriptome assembly offers a significant quality improvement also for the genes which had already been previously detected with pyrosequencing, because of a higher coverage and full-length reconstruction: as a matter of fact, even though most digestive gland contigs find a match in *M. edulis* 454 reads, the detailed analysis of the mapping revealed that about 5,000 contigs displayed very low coverage (<5). This factor, together with the high error rate of pyrosequencing, prevented the reliable reconstruction of many full length mRNAs in Philipp et al. [9], consistently with the lower reported average contig length in their *de novo* assembly (645 bp) compared to our approach (958 bp).

### The digestive gland transcriptome

The global mapping of all Illumina reads obtained from the DG revealed the nuclear genes most typically transcribed in this tissue (Table 3). Not surprisingly, we identified many housekeeping genes such as elongation factor 1 alpha, actin, ferritin and several structural components of the ribosome: their expression is ubiquitous and fundamentally kept stable at very high levels in all cell types and tissues, including the DG, in order to maintain the key cellular functions. Similarly, the high expression of digestive enzymes, such as myosinases and trypsin, is not surprising in this tissue.

**Table 3:** **List of the 30 genes most expressed in the digestive gland of** ***Mytilus galloprovincialis***

Description	Gene length (bp)	Expression (relative to actin)
Unknown	531	5.12
Unknown	486	4.01
Glycine-rich protein	1,021	3.27
Vdg3	501	2.89
Elongation factor 1 alpha	1,82	2.23
Unknown	701	2.02
Myosinase/astacin/meprin-like	1,485	1.88
Putative serine protease inhibitor	368	1.87
Myosinase/astacin/meprin-like	1,453	1.79
Insulin-like growth factor binding protein-like	770	1.70
Vdg3	531	1.69
Putative serine protease inhibitor	479	1.40
Ependemyn-related protein	897	1.28
Ferritin	976	1.28
Ependemyn-related protein	897	1.27
Countin-like protein	1,054	1.18
Trypsin	912	1.12
Ribosomal protein L22	494	1.05
Cathepsin L	1,226	1.04
Unknown	1,374	1.02
HnRNP	673	1.00
Actin	1,884	1.00
Cystatin-like protein	711	1.00
Ribosomal protein S20	556	0.97
Unknown	3,423	0.95
Ribosomal protein S26	570	0.94
Ribosomal protein S15	583	0.89
Unknown	2,151	0.83
Ribosomal protein S2	956	0.82
Ribosomal protein S25	612	0.82

On the contrary, several of the most transcribed genes did not show any similarity with any previously known sequence, suggesting that they may have developed highly specific functions in bivalves. In particular 2 out of the 30 most highly expressed genes pertain to a same family of cysteine-rich peptides, sharing a limited similarity with serine-protease inhibitors. These peptides, together with the highly expressed cystatin, certainly represent key players in this tissue, possibly by regulating the activity of endogenous proteases in the sites of production, avoiding self-digestion.

Interestingly, the two most highly expressed genes (about 4–5 times more than actin) encode short secreted peptides similar with each other, sharing four conserved cysteine residues possibly organized in two disulphide bridges (see Additional file 1) and not showing any similarity to known sequences nor the presence of conserved functional domains. Given their remarkable expression level, these two genes likely play an extremely important role in the digestive gland, but their exact function still remains to be elucidated.

Consistently with previous observations indicating its abundance in DG [8], vdg3 also stands among the genes showing the highest expression levels. Even though the gene function is still unknown, it has been suggested to act as a fundamental regulator of the formation of the juvenile DG [52]. Here we show that vdg3 is present in multiple variants (see Additional file 1), which either suggests the presence of several paralogous genes or of high inter-individual sequence variability. Regardless of the role of vdg3 in early developmental stages, it seems likely that an important digestion-related function is maintained also in mature individuals. Besides the transcripts described above, many other short secreted peptides without similarity and whose function is still completely unknown are expressed at exceptionally high levels in the DG.

Overall, the contigs annotation evidenced a high prevalence of genes encoding proteins with binding properties, and in particular those pertaining to the C1qDC, C-type lectin and FREP families (Table 4), which have a fundamental role in the innate immunity of bivalves [53, 54] and which had already been identified as the most prominent gene families in Mytibase [7]. The expression of such a remarkable number (almost 450) of lectin-like proteins in the DG, a tissue not primarily involved in immune response, further confirms that these gene families have been subject to massive expansion events in bivalves. This abundance is even more impressive considering that the C1q and C-type lectin domains are found more frequently than the most widespread functional domains in the animal kingdom (e.g. calcium binding EF hand, EGF-like, immunoglobulin-like, etc.). Other abundant Interpro annotations, as expected, are obviously linked to the metabolic functions of the DG (e.g. Cytochrome P450, Short-chain dehydrogenase/reductase SDR, Glucose/ribitol dehydrogenase, etc.) and housekeeping processes.

**Table 4:** List of the 30 most abundant InterPro domains in *M. galloprovincialis* digestive gland transcriptome

Interpro domain	Description	Contigs in ***M. galloprovincialis***	Mussel vs ***C. gigas***rate*
IPR001073	Complement C1q protein	232	1.61
IPR001304	C-type lectin	154	1.15
IPR002048	Calcium-binding EF-hand	93	0.80
IPR018378	C-type lectin, conserved site	89	1.64
IPR013032	EGF-like, conserved site	88	0.53
IPR000742	Epidermal growth factor-like domain	83	0.31
IPR018247	EF-Hand 1, calcium-binding site	81	0.74
IPR001680	WD40 repeat	67	0.54
IPR000719	Protein kinase, catalytic domain	63	0.37
IPR017986	WD40-repeat-containing domain	61	0.49
IPR007110	Immunoglobulin-like domain	56	0.45
IPR002290	Serine/threonine-/dual specificity protein kinase, catalytic domain	56	0.90
IPR001507	Zona pellucida domain	54	9.24
IPR000504	RNA recognition motif domain	53	0.91
IPR002198	Short-chain dehydrogenase/reductase SDR	53	1.42
IPR011992	EF-hand-like domain	53	0.43
IPR006703	AIG1	52	3.99
IPR002181	Fibrinogen, alpha/beta/gamma chain, C-terminal globular domain	51	0.58
IPR002347	Glucose/ribitol dehydrogenase	50	1.36
IPR001841	Zinc finger, RING-type	49	0.36
IPR020635	Tyrosine-protein kinase, catalytic domain	48	2.48
IPR000315	Zinc finger, B-box	47	0.17
IPR003582	Metridin-like ShK toxin	44	1.75
IPR002035	von Willebrand factor, type A	44	0.57
IPR017441	Protein kinase, ATP binding site	44	0.44
IPR001881	EGF-like calcium-binding	42	0.58
IPR020683	Ankyrin repeat-containing domain	42	0.27
IPR001128	Cytochrome P450	41	0.68
IPR003599	Immunoglobulin subtype	41	0.50
IPR019775	WD40 repeat, conserved site	41	0.58
IPR002110	Ankyrin repeat	41	0.27

*the rate is the number of annotated contigs in *M. galloprovincialis* divided by the number of *C. gigas* predicted gene models sharing the same annotation.

Interesting conclusions can be drawn from the comparative analysis of domain abundances with the genome of *C. gigas*. Given the incomplete nature of the DG transcriptome, each domain is expected to be found in a lower number in mussel compared to oyster (abundance rate < 1, see Table 4), an organism whose genome has been fully sequenced. Therefore, an over-representation of certain InterPro domains in mussel (abundance rate > 1) is strongly indicative of gene family expansions events. In this respect, the most striking case are transcripts encoding proteins with a zona pellucida domain (abundance rate = 9.2, IPR001507), a protein polymerisation module found at the C-terminus of many secreted glycoproteins with different functions. Another common domain over-represented in mussel is AIG1 (abundance rate = 4, IPR006703), typical of IMAP GTPases, whose function in invertebrates in also unknown. These remarkable differences, which imply large events of gene family expansion, loss and acquisition, find a justification in the quite large genomic divergence among bivalves [3, 4].

### Prevalence of long non-coding RNAs

A relevant fraction of the contigs included in the reference transcriptome did not display any BLAST similarity or annotation (Table 1) and about 4,000 contigs lacked any similarity even to *C. gigas*, *P. fucata* and *L. gigantea* predicted proteins (Additional file 1). While this mainly finds a justification in the still limited representation of bivalve sequences in public databases and in the rather large divergence between *M. galloprovincialis* and oysters, many of the contigs without similarity were also characterized by the absence of an ORF, despite the globally reputed high quality of Illumina sequence data. More specifically, we identified 1,759 sequences (14.6% out of the total) lacking an ORF longer than 50 codons, which were confirmed by a coding potential analysis [55] as strong long non-coding RNA (lincRNA) candidates. While the contigs integrity analysis suggests that a fraction of these sequences may be UTRs of longer fragmented transcripts, several hundred mussel sequences are still expected to be genuine lincRNAs, especially considering their relevant length (113 putative lincRNAs longer than 1Kb were identified).

Although the functional role of most lincRNAs is far from being understood, it is clear that in many cases they can regulate the activity of other genes by natural antisense transcription (NAT), interacting with protein-coding mRNAs either transcribed from the same genomic *locus* by the opposite strand (cis-NAT) or from different *loci* (trans-NAT) [56]. Previous studies evidenced that approximately 10% of the reads obtained from transcriptome sequencing of *Ruditapes philippinarum* were originated by NAT, which therefore seems to be a process occurring with rather high prevalence in bivalves [57]. At the present time, the absence of a reference genome and the use of a non-strand specific RNA-sequencing strategy in this study prevent in-depth analyses on antisense transcription in mussel.

### The case study: effects of toxin accumulation on gene expression in the digestive gland

Concentrations of *A. minutum* varying from 1 to 47 × 10^6^ cells L^−1^ have been reported in toxic blooms [58–62]. We exposed adult *M. galloprovincialis* individuals for five days to 5 × 10^6^ cells L^−1^ of the PSP-producing *A. minutum* AL9T strain, a significant but not extreme concentration selected to simulate mussel PSP contamination at levels comparable to those commonly observed during PSP-producing dinoflagellate blooms (data retrieved from HAEDAT, http://haedat.iode.org/). Potential molecular biomarkers for PSP contamination in the DG were selected according to the following criteria: a) significant responsiveness (either with over- or under-expression) to PSP accumulation b) increased/decreased expression detectable in the early phases of contamination (T1, 24 h) and maintained until the maximal accumulation (T2, 5 days).

Several studies evidenced that the processes of accumulation, transformation and detoxification of PSTs in mussel are characterized by a temporal pattern [63–66]. The physiological early and late/adaptive responses to toxins are likely matched to a parallel alteration of molecular pathways, so the identification of genes specifically regulated at T1 and T2 would potentially provide insights on these still poorly understood mechanisms. However, the lack of RNA-seq replicates and our focus on genes relevant for biomonitorning (and thus differentially expressed both at an early and at a late phase) discouraged this analysis at the present time, even though this is an issue which should be addressed in future studies.

Overall, only 39 contigs met the three previously listed criteria (Table 2). Twenty-eight transcripts were up-regulated, whereas the expression of the remaining 11 significantly decreased in response to the accumulation of paralytic toxins both at T1 and T2. These sequences were selected as the most likely PSP-responsive candidate genes for biomonitoring. The rather low number of transcripts affected and the impossibility of identifying any particular biological process or class of proteins over-represented within this subset is in line with the classification of *Mytilus* spp. as organisms refractory to PSP [35]. More in detail, almost a half of the transcripts of interest did only show similarity with sequences uncharacterized proteins predicted from the genomes of *C. gigas* and *P. fucata* or didn’t show any BLAST similarity at all. In addition, two of the up-regulated contigs were classified as putative lincRNAs due to their low coding potential.

Furthermore, several of the annotated sequences, both among the up- and the down-regulated transcripts, were pertaining to particularly common gene families, i.e. C1q, IMAP GTPases, fibrinogen-related, C-type lectins and zona pellucida domain-containing. Despite the important role of these molecules in many different aspects of mussel life, none of them could be directly linked to functions related to toxin accumulation, excretion, transport or metabolism.

Overall, the expression profiles of contaminated mussels did not point out any indication of massive damages occurring in the DG, and although a few innate immunity related transcripts were differentially regulated (in particular one defensin-like peptide was strongly over-expressed and 2 C1qDC transcripts were down-regulated), no molecular evidence of the massive recruitment of hemocytes and activation of immune defenses reported by other authors [38] could be detected.

A factor which needs to be taken into account is the ability of *Alexandrium* spp. to produce allelopathic extracellular toxins, unrelated to PSPs. These compounds, whose molecular nature is still obscure, are produced to kill nutrient-competing species, but while their negative effects on the planktonic community have been largely documented [67–69], limited attention has been focused so far on their interaction with benthic marine invertebrates. Allelopathic compounds have been hypothesized to be responsible of *A. tamarense* toxicity to grazing gastropod larvae [70], thus evidencing that the spectrum of affected organisms is potentially large, including mollusks, at least at their early life stages. Further evidence raised the possibility that bivalve haemocytes exposed to *Alexandrium* spp. cells or cell extracts may be indeed affected by allelopathic substances of unknown nature [71, 72]. Based on the absence of literature on this topic, we cannot rule out the possibility that both the *A. minutum* strains used in the present study may be producers of molecules other than PSPs somehow toxic to *M. galloprovincialis*, thus potentially masking some of the molecular signatures of PSP response in the RNA-seq experiment.

Therefore, even if the effects of PSTs accumulation on the gene expression in the DG seem to be scarce, given the poor knowledge of the molecular mechanisms linked to phycotoxin accumulation in mussels, our data represent a starting point for future analyses. Due to the low number of animals taken into account in the present study and the absence of biological replicates, the results of the comparisons between experimental conditions clearly need further validation by using a greater number of samples. In addition, the candidate biomarkers of contamination require a direct confirmation in contaminated animals collected during naturally occurring algal blooms.

## Conclusions

The sequencing data generated in this study allowed the global assembly of the *M. galloprovincialis* digestive gland transcriptome. RNA deep sequencing had already been applied to bivalves, but this is the first Illumina technology-based sequencing effort ever reported in *Mytilus*. The resulting transcript sequence collection remarkably improved the sequencing data obtained from previous studies in *Mytilus* spp. and revealed the variety of genes expressed in the digestive gland. Nevertheless, a comprehensive overview of the mussel transcriptome is still far from being reached: only the RNA-seq analysis of additional tissues and vital stages, coupled with strand-specific sequencing strategies will permit to elucidate the complex mechanisms at the basis of the regulation of gene expression in this important bivalve mollusk. In addition, only the availability of a reference annotated genome will permit in the future a comprehensive assessment of several aspects contributing to mussel transcriptomic complexity, including alternative splicing, paralogy and allelic variability. Nonetheless, the new transcriptome assembly provides a valuable resource for improving the molecular knowledge of this species and has already been used as the basis for further studies requiring whole-transcriptome mining approaches [73, 74].

Besides its importance as an improved genetic knowledgebase for mussel genomics, due to its high percentage of full-length mRNAs the reference transcriptome here presented could be used as the basis for gene expression studies focused on the DG, the main tissue involved in the accumulation and biotransformation of xenobiotics in mussel, as highlighted by our case study, which provided the first evaluation of the transcriptional effects of bioaccumulated PSTs in the DG of a bivalve mollusk. Our preliminary results, which still require further validation by the analysis of a larger number of experimental samples, provided the first molecular lines of evidence supporting the classification of mussels as organisms scarcely responsive to PSP, even though a limited number of PSP biomarker genes were identified. Although the occasional reports of PSP adverse effects on mussels [36, 38] did not find confirmation in this study, the different responses described in literature could be linked to inter-population variability in the sensitivity to toxins, in a similar fashion to other mollusk species [75]. Furthermore, our analysis was focused on the DG, the main site of PSTs accumulation, but it cannot be ruled out that other tissues, despite not being directly involved in toxic dinoflagellates digestion, could be heavily affected and reveal better molecular markers of PSP contamination, as suggested by recent studies on oyster haemocytes exposed to PSTs [71, 76].

The identification of a few potential molecular markers typical of PSP contamination could provide the basis for straightforward studies aimed at the development of tools for the biomonitoring of PSP contamination. In particular, the identification of alternative methods is a priority for the monitoring authorities, in order to support the HPLC-based methods [77], and as a strategy to minimize the possibility of PSP contamination in the aquaculture sector [78]. However, in absence of confirmation in naturally exposed mussels, due to the significant inter-individual response differences previously observed in mussels subject to different environmental stressors [79], the high heterozigosity of mussel populations [51], and the low experimental number of individuals per time point (*n* = 3), our data have to be considered as strictly preliminary. Overall, the identification of unequivocal markers of PSP contamination in mussels seems quite a difficult task and certainly requires careful field validation. Such a task will be probably more easily achievable in responsive bivalves, such as oysters and clams, where the remarkable physiological modifications observed are likely matched by evident alteration of gene expression.

## Methods

### Mussel specimens

Adult *Mytilus galloprovincialis* (Lamarck, 1819) specimens were obtained from a commercial producer of the Gulf of Trieste. All the mussels were collected from the same location. Individuals of similar size and weight (medium length 55 ± 4 mm, mean fresh weight, without the shell, 2.48 ± 0.42 g) were acclimated at 15°C and 32‰ salinity for one week in running prefiltered seawater and for 3 days in bacteria-free filtered seawater (Millipore Durapore GV 0,22 μm, hydrophile PVDF) at 12:12 h dark:light regime. Mussels were tested by HPLC before the start of the experiment and were found free of PSP toxins.

### *Alexandrium minutum*cultures

The AL1T (non-toxigenic) and AL9T (toxin producing) strains of *A. minutum*, previously isolated from the Gulf of Trieste, were cultured in medium B [80] in a suitable number of aerated 1 L batch cultures. The cultures were maintained at 15°C at 10:14 h dark:light regime with an irradiance of 60 μE m^−2^ s^−1^. Algal cells were harvested in the late exponential phase of growth.

Both strains were tested at the time points T0, T1 (24 hours) and T2 (5 days) for the production of PSTs as described in the “Toxin analysis” section: 100 ml of culture were filtered on Millipore Durapore GV 0.22 μm filters and immediately frozen at −18°C for HPLC analysis.

### Experimental design

Mussels were maintained in standard conditions in glass tanks containing 0.4 L of 0.22 μm filtered seawater per mussel. Water was renewed every morning at 9 AM with filtered bacteria-free seawater. A total of 6 tanks were prepared for the exposure to *A. minutum*, each one containing 10 mussels. The number of mussels was in surplus in respect with those needed for sampling to face the possibility of mortality during the acclimatization phase. In detail, 3 tanks hosted the AL1T (non-toxigenic) cells and the remaining 3 the AL9T (toxigenic) cells. During the 5 days of intoxication, a dose of 2 × 10^6^ cells of *A. minutum* per mussel was added every 2 hours, 5 times a day, beginning at 10 AM. At selected time points, namely at T1 (24 hours) and T2 (5 days), always at 9.00 AM, one mussel per aquarium was sacrificed for further analyses, thus obtaining 3 biological replicates for each time point and treatment.

### Toxin analysis

The analysis of the PSTs was performed on the *A. minutum* cells and soft mussel tissues at the time point T0, before the first feeding dose, T1 (24 hours after the first feeding) and T2, when the maximum bioaccumulation of toxins was supposedly achieved. The PSTs detection was based on pre-column oxidation and High Performance Liquid Chromatography coupled to Fluorescence Detection (HPLC-FLD) according to the protocol AOAC 2005.06 [81].

The algal pellets were suspended in 0.1 mM acetic acid up to a total volume of 3 mL. The acidic algal suspensions were transferred to a 50 mL centrifuge tube and sonicated for 30 min (sonicator Ultrasonic® Liquid Processor Model XL2020, Heat Systems Inc.) in order to break the algal cells. Sonicated algal suspensions were centrifuged (10 min, 4500 rpm) and aliquots subjected to the analysis.

From each single mussel, whole body tissues deprived of the DG (used in parallel for RNA extraction) were homogenised and tissue aliquots equivalent to 1.7 g were analysed. Following preliminary sample oxidation with both peroxide and periodate, the HPLC-FDL method allows quantitation of individual PSP toxins, with the exception of the epimeric pairs (GTX1\4; GTX2\3, and C1\2) which form identical oxidation products and cannot be separated [82]. Toxins were quantified against linear calibrations of all currently-available PSP toxin certified reference standards and the toxicity equivalence factors (TEFs) proposed by the CONTAM Panel [77] were used to calculate STX-equivalent concentrations and to estimate the concentration of PSTs in the whole mussel tissues.

### RNA extraction and analysis

Digestive glands were excised from the three biological replicates sampled at each of the two selected time points during the exposure to the AL1T and AL9T strains and immediately homogenized in TRIzol® reagent (Life Technologies, Carlsbad, California). Total RNA was individually purified according to the manufacturer’s instructions. Following extraction, the RNA quality was assessed by electrophoresis on denaturing agarose gel and its quantity was estimated by UV-spectrophotometry, based on 260 nm/230 nm and 280 nm/230 nm absorbance ratios (Ultrospec® 2000, Pharmacia Biotech, Bromma, Sweden). RNAs extracted from the 3 biological replicates were pooled in equal quantities and used for the RNA-seq analysis.

### Sequencing and *de novo*reference transcriptome assembly

cDNA libraries were prepared and subjected to massive sequencing at the Biotechnology Center of the University of Illinois, using an Illumina GAII sequencing platform and a 2X100 bp paired-end sequencing strategy. The output sequencing reads were further processed for adapter removal and trimming, according to the base calling quality. The resulting sequences were assembled with Trinity using the default options and a minimum allowed length of 250 bp [83]. The overall quality of the assembly was improved with the addition of 49,871,662 Illumina reads obtained from the DG of naive mussels (unpublished data, Gerdol, Venier and Pallavicini). Finally, we compared the obtained transcriptome to a sequence dataset originated by the assembly of the 18,788 Sanger sequences of Mytibase [7] and 115,557 reads from different tissues of mussels by 454 Life Sciences (unpublished data, Pallavicini and Venier), obtained with the CLC Genomics Workbench assembler. Contigs without a significant BLAST [84] match (considering an e-value threshold of 1 × 10^−50^), representing transcripts poorly expressed in the digestive gland, were added to the overall assembly.

Aiming to obtain a high quality reference transcriptome for the RNA-seq expression analysis and annotation, not subject to random expression fluctuations and excessive fragmentation due to insufficient coverage [85], we only considered contigs displaying a minimum average coverage (25x considering the entire set of Illumina, 454 and Sanger sequences) as reasonably trustworthy and assembled to their full length to the best of the Trinity algorithm technical limitations. Trinity potentially generates multiple contigs for each gene, corresponding to transcripts for alternatively spliced isoforms. Taking this into account, to reduce the redundancy of the assembly prior to the gene expression analysis, only the longest transcripts generated per each gene were annotated and used for the gene expression analysis.

### Contigs annotation and quality assessment

The non-redundant, high quality set of contigs obtained was annotated with the Trinotate pipeline: sequence similarities were identified by BLASTx [86] against the UniProtKB/Swiss-Prot database, functional domains were detected by a HMMER [87] search against the PFAM [88] and InterPro [89] domain databases; finally, eggNOG [90] and Gene Ontology [91] functional categories were assigned. In addition, assembled contigs were compared to the proteins predicted from the genomes of *C. gigas*, *P. fucata* and *L. gigantea* by tBLASTn (using an e-value cutoff of 1 × 10^−5^). The metric used for the assessment of the assembly quality was based on the direct comparison of ortholog length coverage in the fully sequenced genome of *C.gigas* using BLASTx (using an e-value cutoff of 1 × 10^−5^).

The presence of contigs resulting from *A. minutum* RNA contamination were detected by the mapping of the 454 sequencing reads set by Stüken and colleagues [43] on the transcripts reference set with the RNA-seq mapping tool included in the CLC Genomic Workbench v 6.0.5 (Aarhus, Denmark), setting the length and similarity fraction parameters to 0.5 and 0.9, respectively. Contigs originated from mitochondrial and ribosomal RNAs were detected by BLASTn (using NC_006886 and JX081670 as queries, with an e-value cutoff of 1 × 10^−30^) and positive hits were removed from the assembly.

Putative long non-coding RNAs were detected by the absence of an Open Reading Frame (ORF) of at least 50 codons and their coding potential was further assessed with CPC [55].

Based on RNA-seq mapping data (see section below), we investigated the presence and the frequency of SNVs, insertions and deletions, using the “quality based variant detection tool” included in the CLC Genomics Workbench. No gaps and mismatches were allowed in the neighborhood radius (whose value was set to 5). Minimum neighborhood and minimum central qualities were set to 15 and 20, respectively. Only regions displaying coverage higher than 100X were analyzed, and the threshold values for calling a variant were set at 5%, unless a variant was supported by at least 20 read counts.

### Expression analysis by RNA-seq

All the RNA-seq bioinformatics analyses were performed with the tools included in the CLC Genomics Workbench v 6.0.5 (Aarhus, Denmark). Reads obtained from the four samples were mapped to the high quality transcript reference library using the RNA-seq tool, setting the length and similarity fraction parameters to 0.75 and 0.95, respectively. Read counts were normalized with the “normalize” tool, using the scaling method and setting the mean and the median mean as the normalization and reference values respectively, and excluding the lower and upper 5^th^ percentile of the empirical distribution of the expression values from the calculation. Normalized expression values were used for a Kal’s Z-test on proportions statistical analysis to identify differentially expressed transcripts [92].

Comparisons between *A. minutum* AL1T and AL9T strain-fed mussels were performed at T1 and T2 and differential expression was concluded with a Bonferroni-corrected p-value lower than 1 × 10^−10^ and a minimum weighted proportion Fold Change of ± 2. Results were cross-checked in order to select only transcripts significantly differentially expressed at both time points.

## Electronic supplementary material

Additional file 1: **Additional file one contains: the sequence alignments of the predicted protein sequences for the two most expressed transcripts in mussel digestive gland (Additional file** **1** **: Figure S1) and for all the possible isoforms of vdg3 assembled by Trinity (Additional file** **1** **: Figure S2).** - a Venn diagram displaying the overlap between tBLASTn results obtained in *Crassostrea gigas*, *Pinctada fucata*, *Lottia gigantea* and in the whole UniprotKB/SwissProt database (Additional file 1: Figure S3). (DOCX 421 KB)

## Abbreviations

DG: Digestive gland
PSP: Paralytic shellfish poisoning
PST: Paralytic shellfish toxin
NGS: Next generation sequencing
STX: Saxitoxin
UTR: Untranslated region
lincRNA: Long non-coding RNA
ORF: Open reading frame
HAB: Harmful algal bloom.
