# Supplementary material for: RNA sequencing and de novo assembly of the digestive gland transcriptome in Mytilus galloprovincialis fed with toxinogenic and non-toxic strains of Alexandrium minutum
Source: BMC Res Notes. 2014 Oct 14;7:722. doi: 10.1186/1756-0500-7-722 (PMC4203926; doi:10.1186/1756-0500-7-722)
Supplement: Supplementary file 1 — Additional file 1: Additional file one contains: the sequence alignments of the predicted protein sequences for the two most expressed transcripts in mussel digestive gland (Additional file 1 : Figure S1) and for all the possible isoforms of vdg3 assembled by Trinity (Additional file 1 : Figure S2). - a Venn diagram displaying the overlap between tBLASTn results obtained in Crassostrea gigas, Pinctada fucata, Lottia gigantea and in the whole UniprotKB/SwissProt database (Additional file 1: Figure S3). (DOCX 421 KB) [file 13104_2013_3245_MOESM1_ESM.docx]

**BMC Research Notes**

**ADDITIONAL FILE 1**

**RNA sequencing and *de novo* assembly of the digestive gland transcriptome in *Mytilus galloprovincialis* fed with toxinogenic and non-toxic strains of *Alexandrium minutum***

Gerdol Marco^a^, De Moro Gianluca^a^, Manfrin Chiara^a^, Milandri Anna^b^, Riccardi Elena^b^, Beran Alfred^c^, Venier Paola^d^, Pallavicini Alberto^a^*

^a^ Laboratory of Genetics, Department of Life Sciences, University of Trieste, Via Licio Giorgeri 5, 34126 Trieste, Italy

^b^ Fondazione Centro Ricerche Marine, viale Amerigo Vespucci 2, 47042 Cesenatico, Forlì-Cesena, Italy

^c^ Istituto nazionale di Oceanografia e di Geofisica sperimentale, Dipartimento di Oceanografia Biologica, via Auguste Piccard 54, 34151 Santa Croce, Trieste, Italy

^d^ Department of Biology, University of Padua, Via Bassi 58 / B, 35121 Padova (PD), Italy

*corresponding author

Email addresses:

Gerdol Marco: [mgerdol@units.it](mailto:mgerdol@units.it)

De Moro Gianluca: [gdemoro@units.it](mailto:gdemoro@units.it)

Manfrin Chiara: [cmanfrin@units.it](mailto:cmanfrin@units.it)

Milandri Anna: [anna.milandri@centroricerchemarine.it](mailto:anna.milandri@centroricerchemarine.it)

Riccardi Elena: [elena.riccardi@centroricerchemarine.it](mailto:elena.riccardi@centroricerchemarine.it)

Beran Alfred: [aberan@ogs.trieste.it](mailto:aberan@ogs.trieste.it)

Paola Venier: paola.venier@unipd.it

Pallavicini Alberto: [pallavic@units.it](mailto:pallavic@units.it)


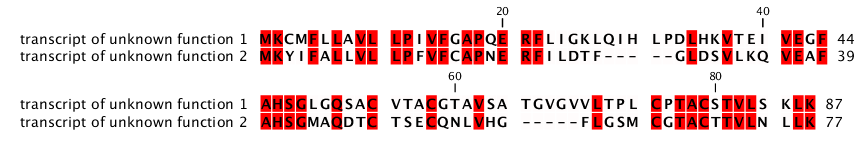


**Supplementary Figure S1**: sequence alignment of the two transcripts of unknown function displaying the highest expression level in the digestive gland. Identical residues are highlighted in red.


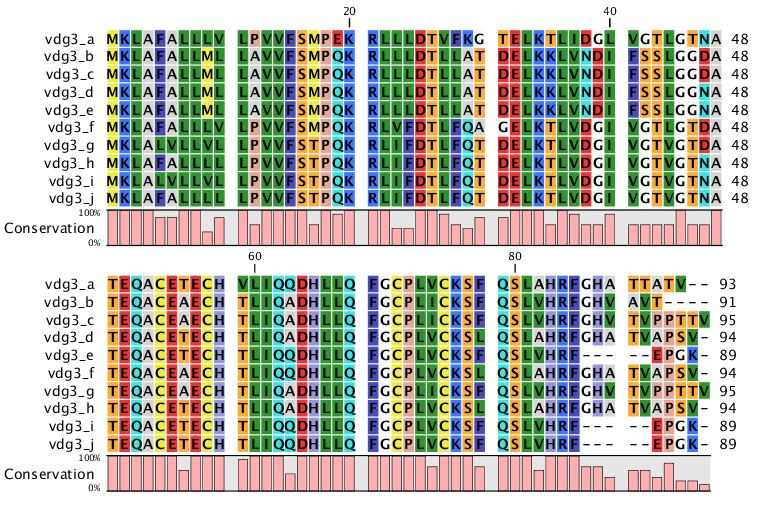


**Supplementary Figure S2**: multiple sequence alignment of all the possible vdg3 isoforms assembled by Trinity.


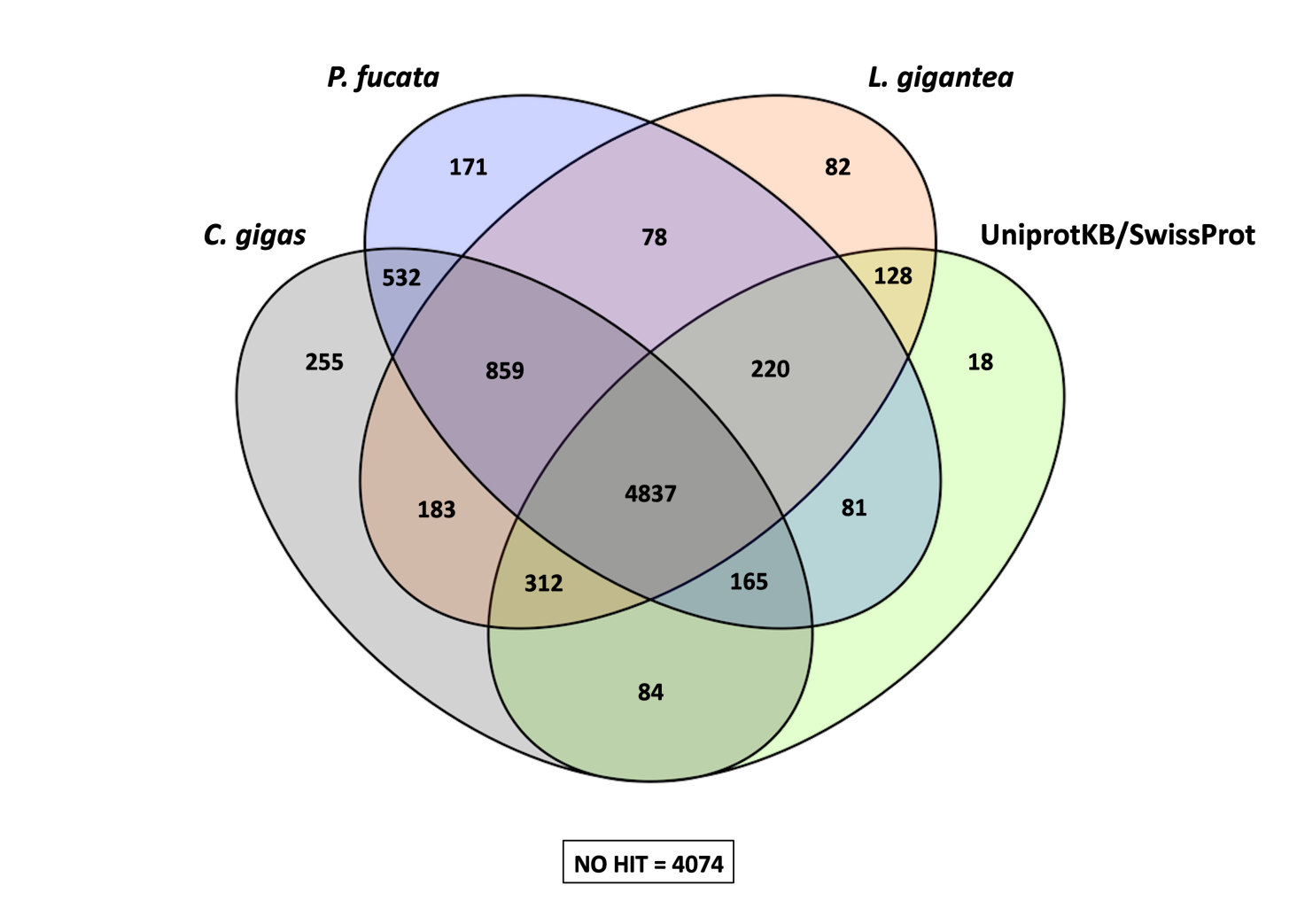


**Supplementary Figure S3**: overlap between tBLASTn results obtained in *Crassostrea gigas*, *Pinctada fucata*, *Lottia gigantea* and in the whole UniprotKB/SwissProt database.
